# Circulating miRNAs: Potential Novel Biomarkers for Hepatopathology Progression and Diagnosis of Schistosomiasis Japonica in Two Murine Models

**DOI:** 10.1371/journal.pntd.0003965

**Published:** 2015-07-31

**Authors:** Pengfei Cai, Geoffrey N. Gobert, Hong You, Mary Duke, Donald P. McManus

**Affiliations:** Molecular Parasitology Laboratory, QIMR Berghofer Medical Research Institute, Queensland, Australia; Wellcome Trust Sanger Institute, UNITED KINGDOM

## Abstract

**Background:**

Schistosomiasis remains a major public health issue, with an estimated 230 million people infected worldwide. Novel tools for early diagnosis and surveillance of schistosomiasis are currently needed. Elevated levels of circulating microRNAs (miRNAs) are commonly associated with the initiation and progression of human disease pathology. Hence, serum miRNAs are emerging as promising biomarkers for the diagnosis of a variety of human diseases. This study investigated circulating host miRNAs commonly associated with liver diseases and schistosome parasite-derived miRNAs during the progression of hepatic schistosomiasis japonica in two murine models.

**Methodology/Principal Findings:**

Two mouse strains (C57BL/6 and BALB/c) were infected with a low dosage of *Schistosoma japonicum* cercariae. The dynamic patterns of hepatopathology, the serum levels of liver injury-related enzymes and the serum circulating miRNAs (both host and parasite-derived) levels were then assessed in the progression of schistosomiasis japonica. For the first time, an inverse correlation between the severity of hepatocyte necrosis and the level of liver fibrosis was revealed during *S*. *japonicum* infection in BALB/c, but not in C57BL/6 mice. The inconsistent levels of the host circulating miRNAs, miR-122, miR-21 and miR-34a in serum were confirmed in the two murine models during infection, which limits their potential value as individual diagnostic biomarkers for schistosomiasis. However, their serum levels in combination may serve as a novel biomarker to mirror the hepatic immune responses induced in the mammalian host during schistosome infection and the degree of hepatopathology. Further, two circulating parasite-specific miRNAs, sja-miR-277 and sja-miR-3479-3p, were shown to have potential as diagnostic markers for schistosomiasis japonica.

**Conclusions/Significance:**

We provide the first evidence for the potential of utilizing circulating host miRNAs to indicate different immune responses and the severity of hepatopathology outcomes induced in two murine strains infected with *S*. *japonicum*. This study also establishes a basis for the early and cell-free diagnosis of schistosomiasis by targeting circulating schistosome parasite-derived miRNAs.

## Author Summary

Schistosomiasis japonica remains a public health problem in Southeast Asia. In China, the number of infected individuals has been reduced considerably due to long-term control efforts over the past 50 years, but still 60 million people are at the risk of the disease. Development of novel tools for early diagnosis and surveillance of schistosomiasis are urgently needed. Circulating microRNAs are increasingly regarded as promising targets for the next generation of diagnostic biomarkers. This study systematically compared both host- and schistosome parasite-derived circulating miRNAs associated with *Schistosoma japonicum* infection in two murine models. For the first time, we revealed that host circulating miRNAs dysregulated after *S*. *japonicum* infection, are mouse strain-dependent, along with different pathological responses. Three host circulating miRNAs, miR-122, miR-21 and miR-34a, may, as a panel, serve as indicative biomarkers for hepatopathology progressions. We also confirmed previous reports of the value of parasite-specific miRNAs (sja-miR-277 and sja-miR-3479-3p) in serum as potential biomarkers for the diagnosis of schistosomiasis japonica. This study establishes a basis for using miRNAs as supplemental biomarkers for the early and cell free diagnosis of schistosomiasis.

## Introduction

Schistosomiasis is a chronic debilitating parasitic disease of humans. Caused by members of the genus *Schistosoma*, it afflicts more than 200 million individuals worldwide, representing a major health and economic burden in tropical and developing nations [1]. The pathology of chronic *Schistosoma japonicum* or *Schistosoma mansoni* infection, in its severe form, results in hepatosplenic schistosomiasis, with clinical symptoms of granuloma formation, periportal fibrosis, portal hypertension, hepatosplenomegaly, ascites, and the formation of vascular shunts [1]. The granuloma formation is characterised by a focussed accumulation of a group of specific immune cells around the schistosome eggs, followed by a fibrosing lesion, which forms as a zone of collagen at its periphery [2,3]. Both features, while beneficial in limiting and neutralising the toxicity of secreted egg antigens (SEA) released from parasite eggs, also cause reversible hepatic damage, indicating that the immune-cellular response is both friend and foe to the schistosome-infected host [4,5].

The general epidemiological situation of schistosomiasis in China has changed due to long-term extensive and integrated control efforts [6,7]. A number of endemic areas are close to transmission interruption and thus improved diagnostic tools are urgently needed for the surveillance of control efforts to ensure that the elimination of schistosomiasis can be achieved [7]. Currently, there are four major methods available for the diagnosis of schistosomiasis: parasitological detection (PD; mainly by the Kato-Katz method), antibody-detection (AbD), antigen-detection (AgD), and the detection of circulating schistosome nucleic acids (CNAD) by PCR. The former three procedures have disadvantages in that they exhibit low sensitivity (e.g. PD), cross-reactivity with other helminth infections or cannot distinguish between active and past infections (e.g. AbD and AgD), with the latter limitation particularly important in endemic areas. The requirement for the detection of schistosome ova in faeces and/or SEA-specific serum antibodies limits such methods for early diagnosis before patency. These inadequacies demonstrate the current limitations in monitoring the progress of schistosomiasis control, especially in areas with low schistosome prevalence or low levels of transmission [8].

MicroRNAs (miRNAs) are a class of small non-coding RNAs approximately 22 nucleotides in length, which can be detected in a wide range of body fluids, including blood plasma/serum [9,10]. The high stability of miRNAs in biofluids has been mainly attributed to two mechanisms: (1) formation of a protein-miRNA complex with argonaute proteins or high-density lipo-proteins, and (2) their incorporation into exosomes [11]. MiRNAs have been increasingly regarded as promising targets for the next generation of diagnostic biomarkers as the strong correlation between the status/progression of various diseases and the dysregulated profile of miRNAs has been confirmed. The potential for detecting circulating miRNAs as biomarkers for various cancers, viral infections, as well as drug-induced liver injury, has been widely reported [9,12–17].

Previously, a panel of host miRNAs was shown to be dysregulated in murine hepatic tissue with the progression of schistosomiasis, highlighting the fact that miRNAs may play a variety of regulatory roles in the immunological responses that occur during the development of hepatopathology [18,19]. The altered expression profile of hepatic miRNAs during schistosomal infection differed from that of other liver diseases [20], indicating that schistosome egg-induced hepatic immunopathology is a unique type of chronic liver disease, distinguishable from many other types of liver disease. Though the diagnostic and therapeutic potential of parasite-derived miRNAs have been discussed [21], the area is still in the early stages of infancy. Nevertheless, five schistosome-specific miRNAs were identified in the plasma of rabbits infected with *S*. *japonicum* using a deep sequencing method and one of them, sja-miR-3479-3p, showed diagnostic potential for *S*. *japonicum* infection [22], although further confirmation in other animal models and in patients is required. Further, the presence of three parasite-derived miRNAs in serum discriminated patients infected with *S*. *mansoni* from normal individuals [18] and circulating parasite-derived miRNAs have been found in the plasma or serum of dogs with a filarial worm infection [23]. Regarding host circulating miRNAs, inconsistent results have been observed in different mouse models of schistosomiasis. For example, the level of liver-specific miR-122 was elevated in the serum of BALB/c mice after *S*. *japonicum* infection [24], while it did not change in the serum of C57BL/6 mice between 4–12 weeks post-*S*. *mansoni* infection [18]. Since these two mouse strains induced differential pathological outcomes, including the severity of hepatic granulomatous pathology and fibrosis at some particular time points post-schistosome infection [25,26], these observations led us to suspect that hepatopathology progression of schistosomiasis may significantly affect the abundance of host miRNAs in serum.

Despite these recent studies, there is generally limited information about the diagnostic value of circulating miRNAs in parasitic diseases and their associated pathologies. We hypothesise that both host- and schistosome parasite-derived miRNAs in serum may present a dysregulated profile during the progression of hepatic schistosomiasis, thereby providing promising targets for an early and cell- diagnosis for the disease. In addition, circulating miRNAs of host origin may provide highly sensitive molecular signatures for the assessment of hepatopathology severity induced by schistosome eggs. Two mouse strains, C57BL/6 and BALB/c mice, were employed to verify our hypothesis.

## Materials and Methods

### Ethics statement

All work was conducted with the approval of the QIMR Berghofer Medical Research Institute Animal Ethics Committee (Ethics Approval: Project P288). Animal studies were conducted according to the Australian Code for the Care and Use of Animals for Scientific Purposes (8th edition) and the protocols approved by the QIMR Berghofer Medical Research Institute Animal Ethics Committee.

### Mice and parasites

Eight-week-old female C57BL/6 and BALB/c mice were percutaneously infected with 14 *S*. *japonicum* cercariae (Chinese mainland strain, Anhui population). Mice were euthanized at 4, 6, 7, 9, 11 (both mouse strains) and 13 (C57BL/6 only) weeks post infection (p.i.). Since BALB/c mice are more susceptible to *S*. *japonicum* infection, the experiment with this strain lasted for 11 weeks p.i. as prolonging the time of infection to 13 weeks would have resulted in premature death of many of the animals due to the resulting egg-induced pathology. Ten naive mice were used as controls for each mouse strain. Each experimental group comprised 10 mice at time points 4, 6 and 7 weeks p.i., and 12 mice were used at 9, 11 and 13 weeks p.i.. The liver and blood samples (~1 mL) were collected by cardiac puncture at each time point. Eggs per gram of liver were calculated as a measure of hepatic egg burden and general infection level, as described [25]. Briefly, eggs were extracted from a portion of liver of known mass by overnight digestion with 5% (w/v) potassium hydroxide. After centrifugation, eggs were then resuspended in 2 mL of 4% (v/v) formalin and the number of eggs in three 10 μL aliquots counted and averaged to calculate the mean eggs per gram of liver (S1 Table).

### Serum collection and RNA extraction

Blood samples were allowed to stand at room temperature for 2 h and then centrifuged at 4,000 rpm for 10 min at 4°C, followed by another centrifugation for 10 min at 10,000 rpm at 4°C. The supernatants were retained and stored at -80°C. Haemolysed samples were excluded from further analysis (S1 Table). For each mouse, RNA was extracted from 100 μL of serum using the miRNeasy mini kit (Qiagen, Hilden, Germany) according to the manufacturer’s protocol. Non-parasitic miRNA (3.2 fmoles), ath-miR-159a, 5′-UUUGGAUUGAAGGGAGCUCUA-3′ (IDT, Coralville, IA) was spiked to each denatured sample to normalize the technical variability of the serum RNA extraction. For each sample, the final RNA product was eluted into 30 μL nuclease-free water and stored at -80°C prior to further analysis. In some experimental groups, blood samples from unisexually infected or uninfected mice without showing any signs of hepatopathology were excluded from further analysis (S1 Table).

### Polyadenylation and reverse transcription (RT)

Polyadenylation and RT reactions were performed with S-Poly(T) method with minor modifications to a published protocol [27]. Total RNA was polyadenylated with a Poly(A) polymerase tailing kit (Epicentre Biotechnologies, Madison, WI) and the first-strand cDNA was synthesized using a TaqMan microRNA reverse transcription kit (Life Technologies, Carlsbad, CA) in a 10 μL RT reaction: 2.53 μL H_2_O, 1 μL 10 × PAP buffer, 0.1 μL ATP (10 mM), 0.5 μL miRNA-specific primer pool (50 nM for each primer), 0.04 μL dNTPs (25 mM each), 0.13 μL RNase inhibitor, 0.2 μL Poly(A) polymerase, 0.5 μL MultiScribe MuLV and 5 μL RNA. Reverse transcription (RT) reactions were conducted using a Veriti 96-well thermal cycler (ABI) under the following conditions: 42°C for 60 min, 95°C for 5 min. RT products were stored undiluted at -20°C prior to the further qRT-PCR reactions. A list of all the primers used in this study is presented in S2 Table. The efficiency of PCR amplification for each primer pair was evaluated by creating a standard curve plot for 10-fold serial dilutions of PCR product (S2 Table).

### Quantification of miRNAs

Quantification of serum miRNAs was performed according to qPCR protocols described previously [28]. Briefly, the 10 μL PCR reaction contained 3 μL H_2_O, 0.5 μL of RT products (2.5× dilution), 0.5 μL forward primer, 0.5 μL universal reverse primer (final conc: 0.2 μM), 0.5 μL universal double-quenched probe (56-FAM/CAGAGCCAC/ZEN/CTGGGCAATTT/3IABkF​Q, final conc: 0.25 μM) (IDT) and 5 μL TaqMan Universal Master Mix II (Life Technologies). Amplification was performed on an Applied Biosystems Viia 7 thermal cycler (Applied Biosystems) with the cycling conditions: pre-denaturation at 95°C for 10 min, followed by 40 cycles: 95°C for 15 sec, and 60°C for 30 sec. For detecting parasite-derived miRNAs, 50 cycles were performed, and the maximum cycle value of 42 was set as background for the purpose of calculating signal over noise. Spiked-in ath-miR-159a was used as the normalized internal control, and the fold change was calculated by the 2^-ΔΔCt^ method [29]. A comparative analysis was carried out to highlight any concordance with respect to host miRNAs between the two mouse strains based on the log base2-transformed qPCR data. The sequences of the primers used are listed in S2 Table. The PCR products were further examined by 15% TBE-PAGE (S1 Fig). Three technical replicates were performed for each sample and repeated PCR assays were carried out for detection of each miRNA (S2 Fig). A biological replicate was carried out with serum samples from BALB/c mice at 4 and 9 weeks post-infection (S3 Fig).

### Histological assessment and biochemical analyses

The median lobe from each mouse liver was used for histological assessment. Formalin-fixed, paraffin embedded liver sections were stained with Haematoxylin and Eosin (H&E) as a measure of granuloma and necrosis, picosirius red for collagen as a measure of fibrosis, alpha-smooth muscle actin (α-SMA) and immunoperoxidase staining for myofibroblasts/Hepatic Stellate Cells (HSCs). Slides were digitised using the Aperio Slide Scanner (Aperio Technologies, Vista, USA). Granuloma volume density and percent of hepatic necrosis, percent of collagen staining (degree of fibrosis) and percent of positive α-SMA staining were quantified with an Aperio ImageScope V10.2.1 with H&E, picosirius red, and α-SMA stained slides, respectively; myofibroblasts/HSCs were defined as α-SMA positive, spindle-shaped cells associated with focal areas of inflammation [25]. Serum alanine transaminase (ALT) and aspartate transaminase (AST) levels were measured with the ALT and AST colour endpoint assay kits (Bioo Scientific, Austin, TX), respectively, according to the manufacturer’s instructions.

### Statistical analyses

All results are reported as means ± SEM (standard error of the mean). For analysis of the serum levels of host miRNAs as well as that of ALT and AST levels during the infection course, one-way ANOVA followed by Holm-Sidak multiple comparison was used. For analysis of relative serum abundance of host miRNAs, hepatic egg burden and histology, two-way ANOVA followed by Holm-Sidak multiple comparisons were used to compare statistical differences between the two mouse strains. For analysis of the parasite-derived miRNAs in serum, the Man-Whitney test was used and p-values of <0.05 were considered statistically significant. Associations were measured using Spearman’s Rho correlation in GraphPad Prism Version 6.00 for windows.

## Results

### Temporal abundance analysis of host serum miRNAs in two murine models during *S*. *japonicum* infection

**Fig 1:**
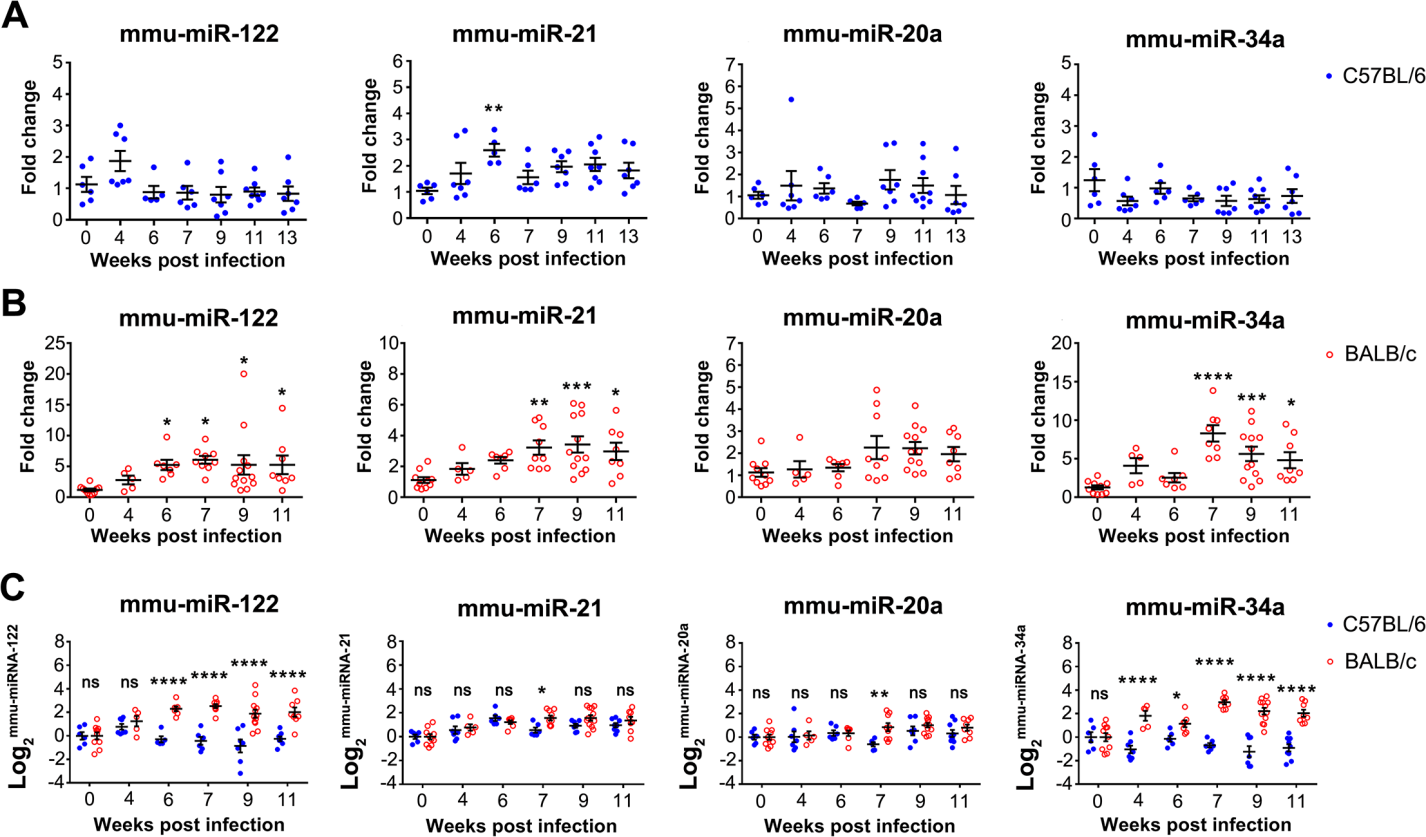
Temporal dysregulation of serum host miRNAs in C57BL/6 and BALB/c mice at different p.i. time points. MiRNAs were quantified by qRT-PCR, normalised to spiked ath-miR-159a, and fold changes were defined as the ratio of serum miRNA abundance in infected mice to naive mice (A, C57BL/6; B, BALB/c). Statistical significance between infected and naive mice was determined using 1-Way ANOVA. Comparisons of relative serum abundance of miRNAs between C57BL/6 and BALB/c mice at 4–11 weeks p.i. were determined using 2-Way ANOVA (C). (* = *P*<0.05, ** = *P*<0.01, *** = *P*<0.001, **** = *P*<0.0001, ns = no significant difference).

Two mouse strains, C57BL/6 and BALB/c, were employed as schistosomiasis japonica disease models to detect in serum, four host circulating miRNAs, miR-122, miR-21, miR-20a and miR-34a, all of which have been suggested to be correlated with different types of liver disease progression [30–33]. In C57BL/6 mice, the serum concentrations of miR-122, miR-20a and miR-34a did not change at any time point post infection, but the level of serum miR-21 was increased at 6 (1-Way ANOVA, *P*<0.01) (Fig 1A). In contrast, apart from miR-20a, the serum levels of the three other host miRNAs were significantly elevated in BALB/c mice by 6 (miR-122) or 7 (miR-21 and miR-34a) weeks p.i. and thereafter (Fig 1B and S3 Fig). Similar results were observed by He *et al*., who found that the levels of miR-122 and miR-34a were significantly elevated in the serum of BALB/c mice at 72 days post-*S*. *japonicum* infection [24]. There was a tendency, albeit not statistically significantly, for relatively high expression of miRNA-20a in the serum of infected BALB/c mice at 7 weeks p.i. and thereafter. This may have been due to the existence of multiple relatively high expressed miRNA-20a isomiRs and the design of the RT-primer against this miRNA, since we only designed one RT-primer against one form of miRNA-20a isomiRs. Thus, the low efficiency of reverse transcription of other miRNA-20a isomiRs might have impaired the sensitivity of detecting this miRNA to some degree. Another explanation is that the expression of miRNA-20a may be down-regulated in the necrotic hepatocytes. Accordingly, we carried out comparative analysis of the relative abundance of these miRNAs in the two mouse strains. With miR-122 and miR-34a, there was a significant difference between the two mouse strains at 4–11 weeks p.i., while for miR-21 and miR-20a, significant difference was only observed at 7 weeks p.i. (Fig 1C). In light of these differing results between the two strains, we carried out further parasitological, histological and serum chemistry analyses.

### Parasitological, histological and serum biochemistry analyses

**Fig 2:**
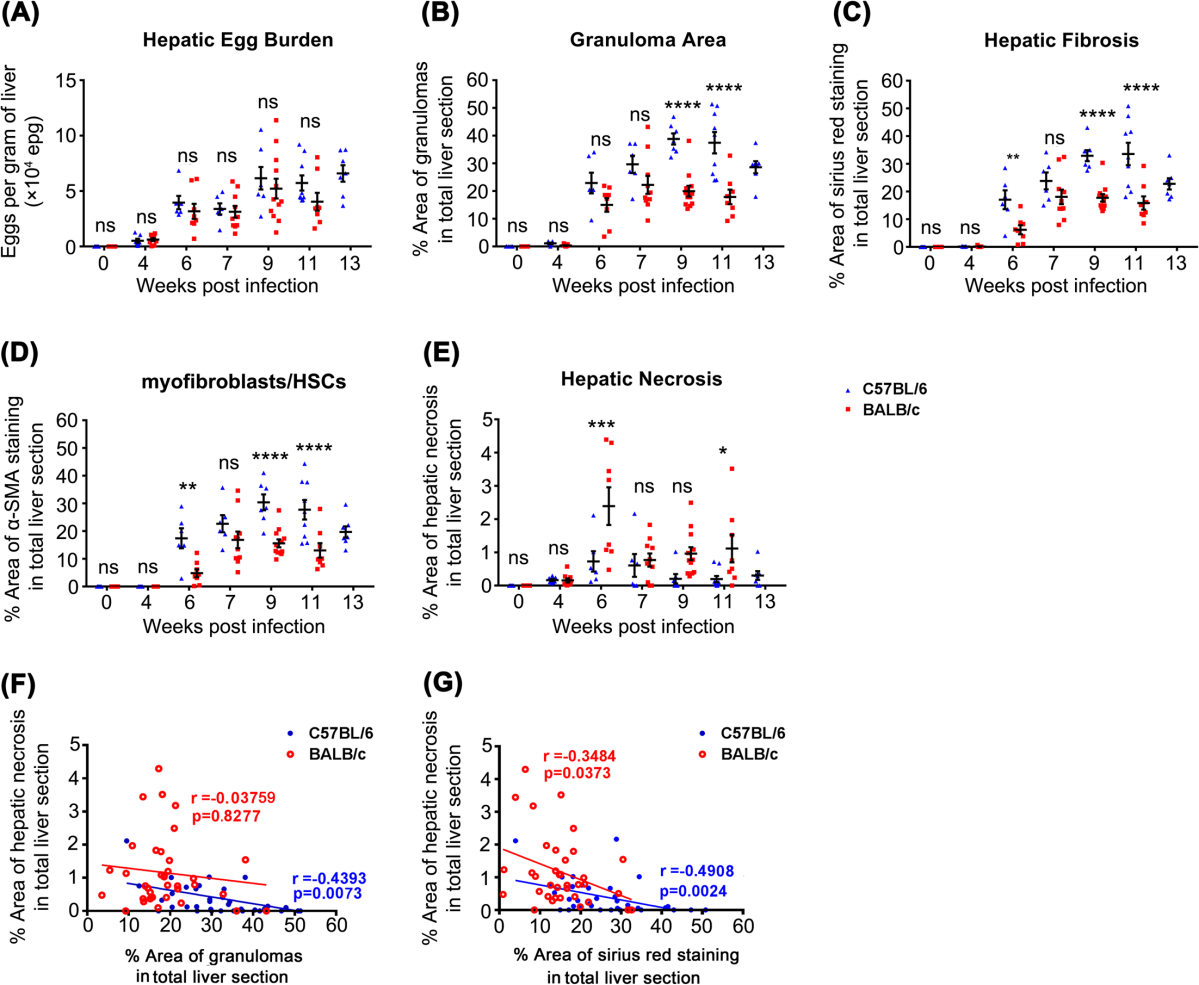
Parasitological and histological comparisons between C57BL/6 and BALB/c mice during *S*. *japonicum* infection. Hepatic egg burdens (A) did not differ significantly between strains; Granuloma area (B), Hepatic fibrosis (C), and myofibroblasts/Hepatic Stellate Cells (HSCs) numbers (D) were significantly increased in C57BL/6 mice compared with BALB/c mice at particular time points p.i.; Hepatic necrosis (E) was significantly more extensive in BALB/c mice compared with C57BL/6 mice at 6 and 11 weeks p.i.. Statistical significance between strains was determined using 2-Way ANOVA. (* = *P*<0.05, ** = *P*<0.01, *** = *P*<0.001, **** = *P*<0.0001, ns = no significant difference). Correlations between the severity of liver necrosis and the level of hepatic granuloma (F), as well as the level of hepatic fibrosis (G) in both mouse strains were performed using Spearman’s Rho correlation.

There were no significant differences in hepatic egg burden between the two mouse strains at any time-point (2-Way ANOVA, *P*>0.05) (Fig 2A), indicating that differences in hepatic egg burden did not cause the differential serum levels of miR-122, miR-21 and miR-34a observed in the two mouse strains during *S*. *japonicum* infection.

**Fig 3:**
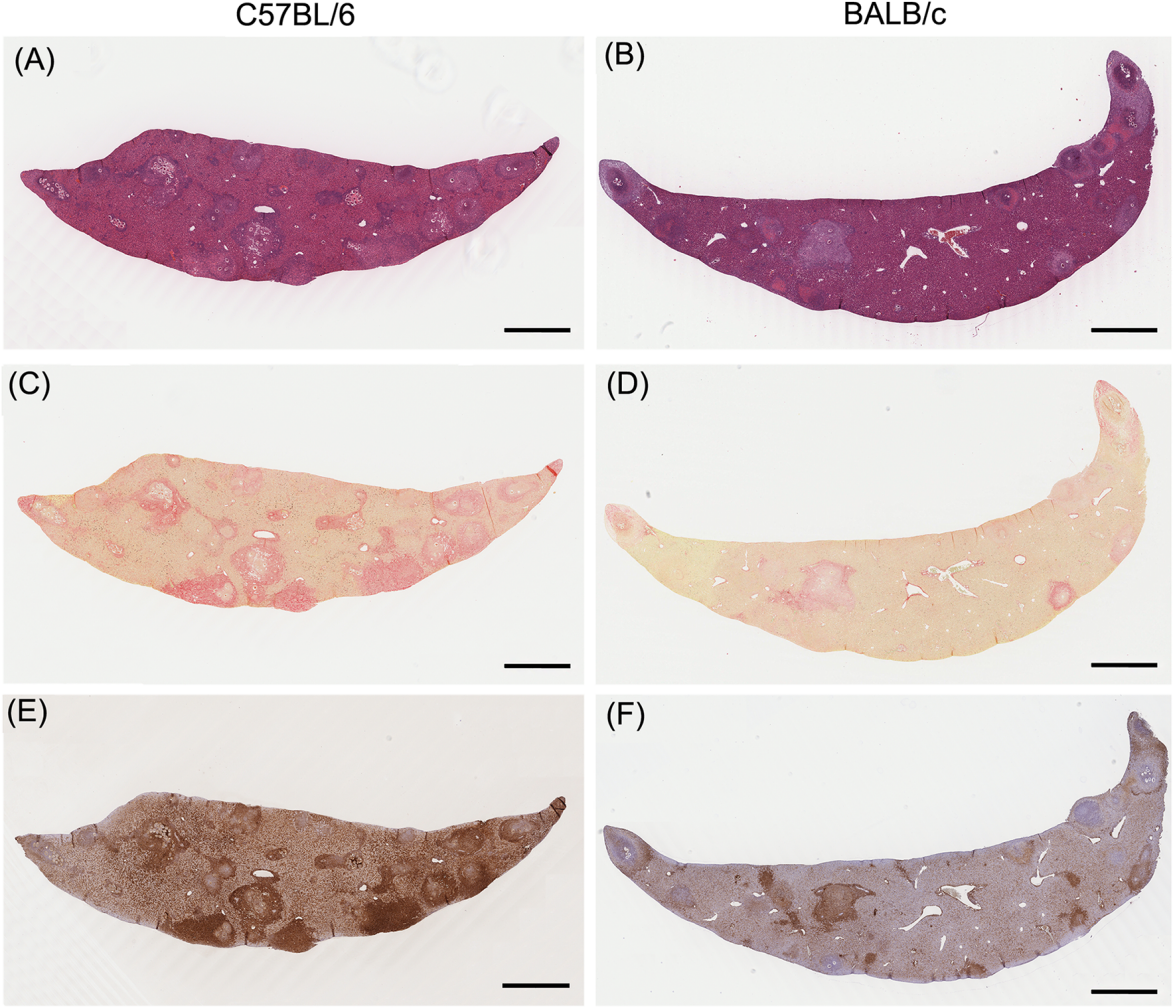
Histological staining demonstrating differences in hepatopathology between C57BL/6 and BALB/c mice. Granulomatous pathology was more severe in C57BL/6 mice; in contrast, hepatic necrosis (pink) was more intensive in BALB/c mice (A and B; Haematoxylin and Eosin). Collagen deposition (red) was also greater in the C57BL/6 mice (C and D; Sirius Red), as was the activation of myofibroblasts/HSCs (brown) (E and F; α-SMA staining). All images were derived from mice at 6 weeks p.i. and were taken from murine livers with similar egg burdens. A, C and E (C57BL/6); B, D and F (BALB/c) were taken from liver sections of the same mouse. Scale bar = 1 mm.

Granuloma area was significantly greater in infected C57BL/6 mice compared with BALB/c mice at 9 and 11 weeks p.i.. In C57BL/6 mice, the granuloma area represented 38.8% and 37.5% of the total liver area at 9 and 11 weeks p.i., respectively, whereas the granuloma area in BALB/c mice was 19.9% and 17.9% of the total liver area at the corresponding time points, respectively (2-Way ANOVA, *P*<0.0001) (Fig 2B). These differences are likely due to the hepatic granuloma area being continually increased in size in C57BL/6 mice between 7~9 weeks p.i., while it had plateaued by this time in BALB/c mice. Although not statistically significant, granuloma area also showed a tendency towards increased size in C57BL/6 mice compared with BALB/c mice at 6 weeks p.i. (Fig 3A and 3B).

Hepatic fibrosis was induced more rapidly in C57BL/6 mice compared with BALB/c mice. This was reflected by significantly greater collagen deposition in C57BL/6 mice at 6 weeks p.i., where collagen represented 17.1% of the total liver area, compared with 6.2% in BALB/c mice (2-Way ANOVA, *P*<0.01) (Figs 2C, 3C and 3D). However, at 7 weeks p.i. there was no significant difference in hepatic fibrosis between the two mouse strains (2-Way ANOVA, *P*>0.05). Similar to the granuloma area, hepatic fibrosis in C57BL/6 mice was significantly more intensive than those in BALB/c mice at 9 and 11 weeks p.i. (2-Way ANOVA, *P*<0.0001). The differential activation level of myofibroblasts/Hepatic Stellate Cells (HSCs) in the two mouse strains showed a similar pattern with hepatic fibrosis (Figs 2D, 3E and 3F).

In contrast, H&E staining indicated that hepatic necrosis was significantly more pronounced in BALB/c mice than in C57BL/6 mice at 6 and 11 weeks p.i. (2-Way ANOVA, *P*<0.001 and *P*<0.05, respectively) (Fig 2E). It is noteworthy that, on average, the necrosis area represented 0.73% of the total liver area in C57BL/6 mice, whereas it reached 2.39% in BALB/c mice at 6 weeks p.i. (Figs 2E, 3A and 3B). Followed the development of granulomas and liver fibrosis, the severity of hepatic necrosis was alleviated in BALB/c mice and no significant differences were observed between the two mouse strains at 7 and 9 weeks p.i.. Based on the analysis of data from 6–13 weeks p.i., a significant inverse correlation was observed between the level of hepatic necrosis and the degree of granuloma formation in C57BL/6 mice (r = -0.4393, *P* = 0.0073), but not in BALB/c mice (Fig 2F). More importantly, significant inverse correlations were observed between the level of hepatic necrosis and fibrosis in both mouse strains (C57BL/6, r = -0.4908, *P* = 0.0024; BALB/c, r = -0.3484, *P* = 0.0373) (Fig 2G). These observations led us to hypothesise that the up-regulation of serum miR-122, miR-21 and miR-34a levels in BALB/c mice during infection may be mainly due to the massive release of these miRNAs from necrotic hepatocytes.

**Fig 4:**
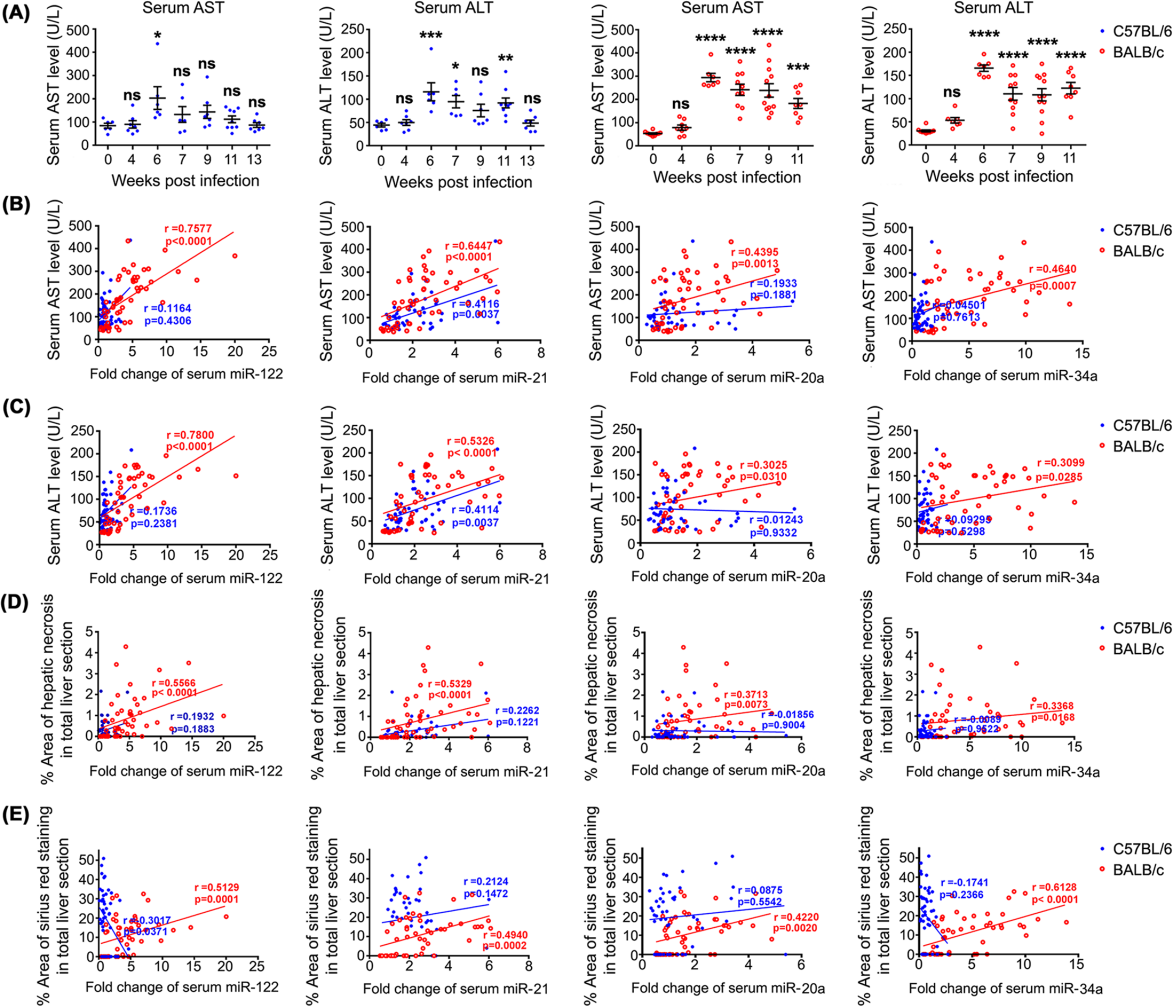
Temporal changes in serum AST and ALT activities in C57BL/6 and BALB/c mice at different p.i. time points and correlation analysis between the serum levels of miRNAs, liver enzymes and the severity of hepatopathology. Serum AST and ALT activities were more dramatically up-regulated in BALB/c mice than in C57BL/6 mice during *S*. *japonicum* infection (A). Statistical significance between infected and naive mice was determined using 1-Way ANOVA. (* = *P*<0.05, ** = *P*<0.01, *** = *P*<0.001, **** = *P*<0.0001, ns = no significant difference). Correlations between the serum levels of miRNAs and AST level (B); ALT level (C), the severity of liver necrosis (D) and hepatic fibrosis (E) in both mouse strains were performed using Spearman’s Rho correlation.

In order to test this hypothesis, we further examined the dynamic changes in serum levels of the liver injury-related enzymes, aspartate aminotransferase (AST) and alanine aminotransferase (ALT). Similar to the temporal alteration of the serum level of miR-21 in C57BL/6 mice, the serum AST level was only significantly elevated at 6 weeks p.i. (1-Way ANOVA, *P*<0.05) (Fig 4A), whereas the serum ALT level was significantly up-regulated at 6, 7 and 11 weeks p.i. (1-Way ANOVA, *P*<0.001, *P*<0.05, and *P*<0.01, respectively). In BALB/c mice, both serum AST and ALT levels were significantly elevated at 6 weeks p.i. and thereafter (1-Way ANOVA, *P*<0.0001, except for AST level at 11 weeks p.i., *P*<0.001). Further analysis revealed that the serum levels of all four miRNAs were significantly correlated with ALT and AST levels in BALB/c mice (Fig 4B and 4C), whereas only the serum level of miR-21 showed a significant correlation with the serum levels of these liver enzymes in C57BL/6 mice (Fig 4B and 4C). Moreover, as shown in Fig 4D, the serum levels of all four miRNAs were significantly positively correlated with the severity of liver necrosis in BALB/c, but not in C57BL/6 mice, an observation which provided additional direct evidence in support of our hypothesis. The reason why serum miRNA levels did not correlate with the severity of liver necrosis in C57BL/6 mice is largely because the degree of hepatic necrosis in this strain is not as pronounced as in BALB/c mice during the course of *S*. *japonicum* infection. Further, the abundance of serum miRNAs in C57BL/6 mice are at baseline levels post-infection. Among these four miRNAs, the serum concentration of miR-122 showed the strongest association with the serum levels of liver injury-related enzymes and the severity of hepatic necrosis in BALB/c mice during *S*. *japonicum* infection, and this was followed by miR-21 (Fig 4B–4D). In addition, the abundance of serum miR-34a showed the strongest association with the degree of liver fibrosis in BALB/c mice during schistosomiasis progression, followed by miR-122 and miR-21 (Fig 4E).

### Detection of parasite-derived miRNAs in the serum of C57BL/6 and BALB/c mice during *S*. *japonicum* infection

**Fig 5:**
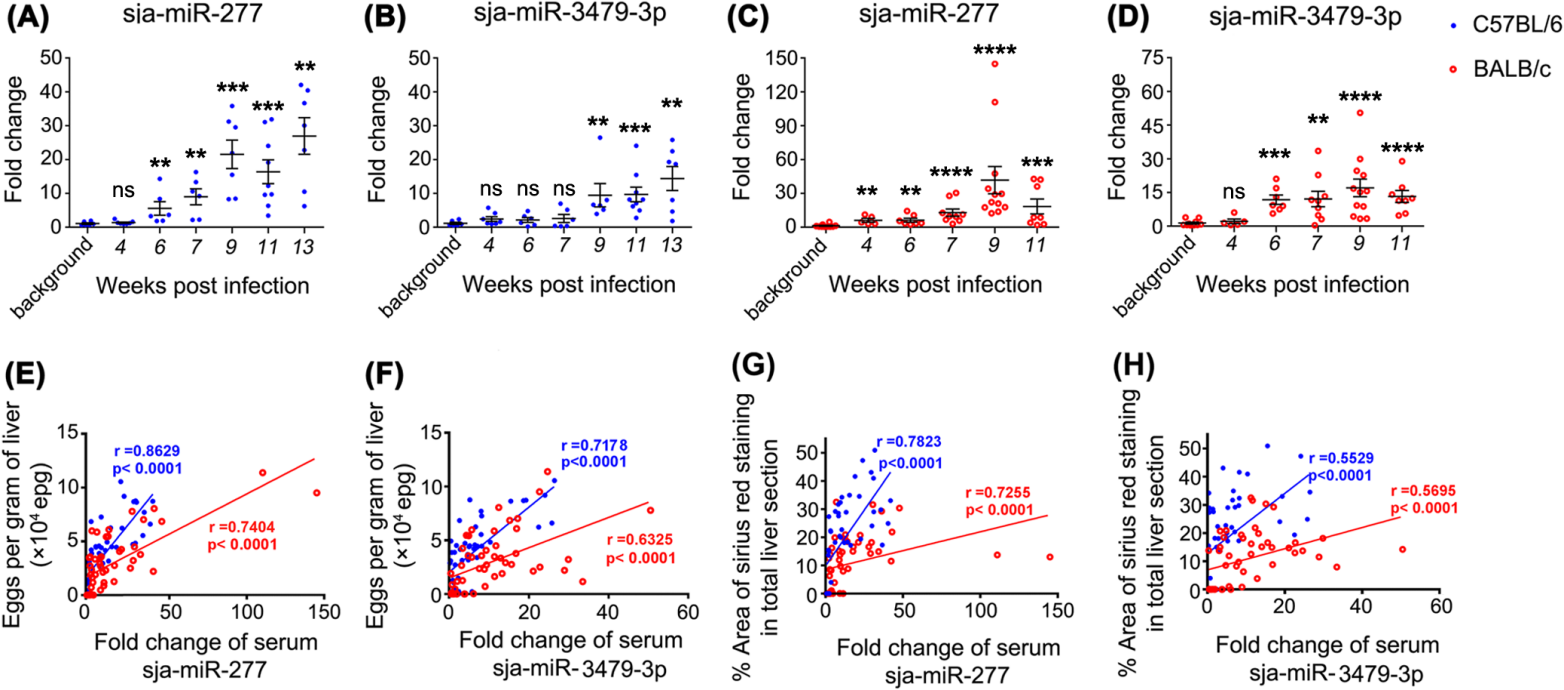
Temporal serum levels of parasite-derived miRNAs in C57BL/6 and BALB/c mice at different time points and their correlations with hepatic egg burdens. The serum levels of sja-miR-277 and sja-miR-3479-3p in C57BL/6 (A and B) and BALB/c (C and D) mice, respectively, during the course of infection. MiRNAs were quantified by qRT-PCR and normalised to the spiked ath-miR-159a; fold changes are defined as the ratio of miRNA abundance in the serum of infected mice compared with the background level in the serum of naive mice. Statistical differences between infected and naive mice were determined using the Man-Whitney test (ns = no significant difference, ** = *P*<0.01, *** = *P*<0.001, **** = *P*<0.0001). Correlations between the serum level of sja-miR-277/sja-miR3479-3p and hepatic egg burden (E and F), as well as the degree of hepatic fibrosis (G and H) were performed for both mouse strains.

Using a deep sequencing method, Cheng *et al*. identified the presence of five schistosome-specific miRNAs (sja-bantam, sja-miR-3479-3p, sja-miR-10-5p, sja-miR-3096 and sja-miR-8185) in the plasma of *S*. *japonicum*-infected rabbits. Three of these (sja-bantam, sja-miR-3479-3p, sja-miR-10-5p) were further detected in the plasma of *S*. *japonicum*-infected mice by stem-loop RT-PCR analysis [22]. More recently, it was shown that *S*. *mansoni*-derived miRNAs (miR-277, miR-3479-3p and bantam) in serum could discriminate infected from uninfected individuals [18]. We thus carried out RT-qPCR analysis to determine the dynamic serum levels of five parasite-derived miRNAs (sja-bantam, sja-miR-3479-3p, sja-miR-3096, sja-miR-8185 and sja-miR-277) during *S*. *japonicum* infection. Sja-miR-10-5p was excluded from analysis due to its high sequence homology with mammalian host orthologs. Only two parasite-derived miRNAs (sja-miR-277 and sja-miR-3479-3p) could be reliably detected in serum specimens from both mouse strains (Figs 5A–5D and S3 Fig). However, one intriguing feature was the time phase when the serum levels of these two miRNAs started to significantly alter differed in the two models. In BALB/c mice, sja-miR-277 and sja-miR-3479-3p showed a statistically significant signal over noise as early as 4 and 6 weeks p.i. (Fig 5C and 5D), respectively, while in C57BL/6 mice, these signals were delayed to 6 and 9 weeks p.i. (Fig 5A and 5B), respectively. Moreover, both the serum levels of sja-miR-277 and sja-miR-3479-3p were significantly correlated with hepatic egg burdens (Fig 5E and 5F) and the degree of liver fibrosis (Fig 5G and 5H) in the two mouse strains. However, the serum level of sja-miR-277 showed a stronger correlation with liver fibrosis intensity than that of sja-miR-3479-3p. All key results obtained with the two mouse strains are summarized in Table 1.

**Table 1:** Summary of key findings comparing C57BL/6 and BALB/c mice during *S*. *japonicum* infection.

	C57BL/6	BALB/c
**Serum chemistry**		
Changes in serum AST level	+	++++
Changes in serum ALT level	++	++++
**Hepatic histology**		
Hepatic granuloma	+++	+
Hepatic fibrosis	+++	+
Hepatic necrosis	+	++
**Host circulating miRNAs**		
Changes in serum mmu-miR-122 level	-	+++
Changes in serum mmu-miR-21 level	+	+++
Changes in serum mmu-miR-20a level	-	-
Changes of serum mmu-miR-34a level	-	+++
**Parasite-derived miRNAs**		
Changes in serum sja-miR-277 level	++, 6 weeks p.i.*	+++, 4 weeks p.i.*
Changes in serum sja-miR-3479-3p level	+, 9 weeks p.i.*	++, 6 weeks p.i.*

* The time point when the circulating miRNA started to show a significantly different level in the *S*. *japonicum*-infected mice compared with naïve mice.

## Discussion

The recent discovery of the extreme stability of circulating miRNA in body fluids and the fact their dysregulated profiles are associated with disease progression are characteristic of a wide variety of diseases and syndromes. These features of miRNAs have trigged widespread interest in their potential as biomarkers for diagnosis and pathologic status of chronic and infectious diseases. This study aimed to investigate whether several circulating host miRNAs commonly associated with liver diseases are dysregulated in murine schistosomiasis japonica and whether *S*. *japonicum*-derived miRNAs could be detected in serum specimens from two mouse strains during disease progression, thus determining their potential value as biomarkers for evaluation of the severity of hepatopathology caused by schistosome eggs and the detection of *S*. *japonicum* infection, respectively.

The advantage of using two mouse strains in this study was the capacity to observe the considerably different dysregulation of circulating host miRNAs, miR-122, miR-21 and miR-34a, in the sera of C57BL/6 and BALB/c mice during *S*. *japonicum* infection. We also assessed the fibrogenic granulomatous response induced by schistosome eggs which leads to the hepatopathology characteristic of schistosomiasis, an area of considerable interest [5,34–37]. In contrast, hepatic necrosis is another type of hepatopathology caused by the toxicity due to schistosome eggs, an area which has received much less attention. The key differences in hepatopathology observed between the two mouse strains examined here is centrally linked to the delayed development of fibrosis, with the level of hepatic necrosis in BALB/c mice markedly more extensive than in C57BL/6 mice at 6 weeks p.i.. The different degree of leakage of egg antigens into the adjacent liver tissue at 6 weeks p.i. in the two mouse strains may contribute partially to the intensity of fibrosis latter as soluble egg antigen (SEA) of schistosomes has been shown to suppress the activation and facilitate apoptosis of HSCs [37,38]. For the first time, we have shown an inverse correlation between the severity of hepatocyte necrosis and the level of liver fibrosis in both mouse models, which further supports the protective role of fibrosis in restricting the SEA within focal areas of chronic inflammation, thus reduce the hepatotoxic effects caused by the eggs trapped in the liver tissue [39]. Also this may explain the differential serum levels of hepatocellular enzymes, as well as different abundances in some serum host miRNAs observed between the two mouse strains. Thus, as summarized in Table 1, the differential levels of miR-122, miR-21 and miR-34a in host sera are mainly the result of hepatopathology caused by the different types of immune response induced in C57BL/6 and BALB/c mice after *S*. *japonicum* infection, especially following the onset of egg deposition. These three host circulating miRNAs may, as a panel, serve as indicative biomarkers for the severity of hepatopathology outcomes, particularly regarding hepatocytes damage or necrosis, in patients having similar worm burdens. However, further studies with clinical samples are needed to justify this suggestion.

It is well known that circulating miRNAs are derived either via passive release of cellular contents from tissue damage or via active secretion of microvesicles/exosomes from cells [40]. Here, significant correlations between the levels of serum miRNAs (miR-122 and miR-21) and liver enzymes indicate that the passive release from injured tissues may represent a key mechanism for the observed increased levels of these miRNAs. MiR-122 is the predominant liver-specific miRNA, constituting about 70% of the total miRNA population in normal liver tissue [41]. This may explain why the significant alteration in miR-122 serum levels could be sensitively detected in BALB/c mice as early as 6 weeks p.i., at the same time when hepatic necrosis is evident. There are consistent observations that miR-122 serum levels are elevated in a number of liver diseases with different etiologies, suggesting that this miRNA may act as a clear biomarker of general liver injury [11,42]. Furthermore, plasma miR-122 has been shown to have a better performance than ALTs in the detection of liver injury [43,44]. The serum miR-20a and miR-34a levels showed a significant but weaker correlation with the serum AST and ALT levels than those of miR-122 and miR-21 in BALB/c mice. These data indicate that other tissues, such as the spleen and/or intestine, which also retain schistosome eggs, may contribute to the serum levels of miR-20a and miR-34a, both are multi-tissue expressed miRNAs [45,46]. It would be useful to investigate the pathology of other injured organs during schistosomiasis for the complete recognition of potential sources of these new biomarkers.

In BALB/c mice, the elevated serum miR-122 and miR-21 levels showed a much stronger correlation with hepatocellular enzymes than with the level of hepatic necrosis. This can be explained by the fact that hepatic necrosis peaked at 6 weeks p.i. in this strain and dramatically decreased thereafter, while the serum levels of miR-122 and miR-21 reached a plateau after 7 weeks p.i., due to accumulation of these miRNAs, which are extremely stable in body fluids [9,47]. Meanwhile, the degree of hepatic granuloma and fibrosis also stabilized after 7 weeks p.i. in BALB/c mice, which resulted in significant positive correlations between the serum miR-122 and miR-21 levels and hepatic fibrosis severity. However, no positive correlation in the serum levels of these four miRNAs and the degree of liver fibrosis was observed in C57BL/6 mice. Hence, it could be misinterpreted that the elevation in serum levels of miR-122 and miR-21 was caused by the fibrogenic granulomatous responses, rather than actually being caused by necrosis, if only the results from BALB/c mice were considered. Previously, He *et al*. showed that circulating miR-223 could serve as a potential novel biomarker for the detection of *S*. *japonicum* infection [24]. However, miR-21, miR-122 and miR-223 were also shown elevated in the serum of patients with HCC (hepatic cellular carcinoma) and chronic hepatitis and these miRNAs were suggested as novel biomarkers for liver injury but not specifically for HCC [32], thereby providing support that the elevation of serum miRNA-223 level might also be caused by liver necrosis due to *S*. *japonicum* infection.

Three circulating *S*. *mansoni*-derived miRNAs, sma-miR-277, sma-miR-3479-3p and sma-bantam, have been shown to have potent diagnostic value in detecting S. *mansoni* infection [18], but in the current study, only two orthologs, sja-miR-277 and sja-miR-3479-3p, could be reliably detected in the sera of the two mouse strains infected with *S*. *japonicum*. This may be due to two reasons: (1) mice were challenged with a low dose of cercariae compared with the previous study [18]; (2) the serum level of sja-bantam was comparatively lower than that of sja-miR-277 and sja-miR-3479-3p. We detected significant increases in the serum level of sja-miR-277 in BALB/c and C57BL/6 mice at 4 and 6 weeks p.i., respectively, earlier than with *S*. *mansoni—*8 weeks p.i.-, although a higher challenge was given in their study. This could have a technical explanation that, unlike Hoy *et al*., who employed the miScript system to perform the reverse transcription reactions [18], here, we employed the S-Poly(T) method, which has been shown to improve both the specificity and sensitivity of the PCR reaction [27]. As miRNAs usually have different isoforms, known as isomiRs [48,49], the design of multiple RT primers against different isomiRs originated from one particular miRNA may further improve the sensitivity of miRNA detection when using the S-Poly(T) method.

We found that schistosome egg-induced pathology may also impact on the detection of parasite-derived miRNAs in serum. This was reflected by the differential time phase for detecting sja-miR-277 and sja-miR-3479-3p in serum. Hepatic granulomas and more importantly, fibrosis, tightly enclose most schistosome eggs trapped in the liver, so as to limit the release of the parasite-derived hepatotoxic proteins and miRNAs from eggs into the surrounding tissue. This may have delayed the detection of these miRNAs in the serum of C57BL/6 mice, when compared with BALB/c mice. The significant correlation between the serum level of these parasite miRNAs and the hepatic egg burden indicates that eggs might serve as an important source for these miRNAs, since a single adult worm pair of *S*. *japonicum* can release an estimated number of 3,000 eggs per day. It is also notable that sja-miR-277 and sja-miR-3479-3p are not the most highly expressed miRNAs in either adult worms or eggs, but are observed at intermediate levels [50]. The most highly expressed miRNAs in the parasite, such as sja-miR-71, sja-miR-71b-5p and sja-miR-1 [50], are undetectable in the serum/plasma of animal hosts infected with schistosomes [18,22]. This observation suggests that schistosome parasite-derived miRNAs, which may be expressed in a cell- or tissue-specific pattern, are selectively released by adult worms and eggs. Exosomes display significantly different selective enrichment of specific extra-cellular miRNAs compared to those from their source cells [51]. However, it does not exclude the fact that adult worms secrete miRNAs into the circulating blood stream, since we have also detected sja-miR-277 and sja-miR-3479-3p in the serum of mice infected with unisexual male worm(s) (S4 Fig). Thus, adult worms and/or eggs contribute to the origin of these parasite-derived miRNAs in serum, which may represent potential markers for the early diagnosis of schistosomiasis.

In summary, inconsistent serum levels of host miR-122, miR-21 and miR-34a in different murine models during infection may impair their value as diagnostic biomarkers for schistosomiasis. However, the serum levels of these miRNAs as a panel may correlate with the hepatic immune responses of a schistosome-infected individual, and they may serve as novel biomarkers to indicate the degree of hepatopathology caused by schistosomiasis. The circulating parasite-specific miRNAs, sja-miR-277 and sja-miR-3479-3p, have potential to be diagnostic markers for schistosomiasis japonica, but the sensitivity for early detection of these miRNAs may not only depend on the parasite load but may also be affected by the host pathology induced by schistosome eggs.

## Supporting Information

S1 Fig The specificity of the PCR products was validated by 15% TBE-PAGE gels. M, Ultra low range DNA ladder; lane 1, ath-miR-159a; lane 2, mmu-miR-122; lane 3, mmu-miR-21; lane 4, mmu-miR-20a; lane 5, mmu-miR-34a; lane 6, ath-miR-159a; lane 7, sja-miR-277; lane 8, sja-miR-3479-3p.
(TIF)

S2 Fig Pearson correlation scatter plots of the levels of serum miRNAs in C57BL/6 (A) and BALB/c (B) mice between the two replicate qRT-PCR assays. Pearson correlation values vary between 0.7375 and 0.9335.
(TIF)

S3 Fig Data obtained from the repeat experiment with BALB/c mice at 4 and 9 weeks p.i. (A) Hepatic egg burdens; (B) Serum AST and ALT levels. Statistical significance between infected and naive mice was determined using 1-Way ANOVA (ns = no significant difference, *** = *P*<0.001, **** = *P*<0.0001). (C) Serum levels of host miRNAs (mmu-miR-122, mmu-miR-21, mmu-miR-20a and mmu-miR-34a); fold changes are defined as the ratio of serum miRNA abundance in infected mice compared with naive mice. Statistical significance between infected and naive mice was determined using 1-Way ANOVA (ns = no significant difference, * = *P*<0.05, ** = *P*<0.01); (D) Serum levels of parasite-derived miRNAs (sja-miR-277 and sja-miR-3479-3p); fold changes are defined as the ratio of miRNA abundance in the serum of infected mice compared with the background level in the serum of naive mice. Statistical significance between infected and naïve mice was determined using the Man-Whitney test (ns = no significant difference, * = *P*<0.05, *** = *P*<0.001).
(TIF)

S4 Fig The serum levels of parasite-derived miRNAs, sja-miR-227 (A) and sja-miR-3479-3p (B) in C57BL/6 mice infected with unisexual male worm(s). Fold changes are defined as the ratio of miRNA abundance in infected mice serum compared with the background abundance level in naive mice serum. Statistical significance between infected and naïve mice was determined using the Man-Whitney test (** = *P*<0.01).
(TIF)

S1 Table Hepatic egg burden values for each mouse and reasons for sample exclusion. (XLSX)

S2 Table Sequences of oligonucleotide primers and probe used in this study. (XLSX)
