# Supplementary figures and images for: Circulating miRNAs: Potential Novel Biomarkers for Hepatopathology Progression and Diagnosis of Schistosomiasis Japonica in Two Murine Models
Source: PLoS Negl Trop Dis. 2015 Jul 31;9(7):e0003965. doi: 10.1371/journal.pntd.0003965 (PMC4521869; doi:10.1371/journal.pntd.0003965)

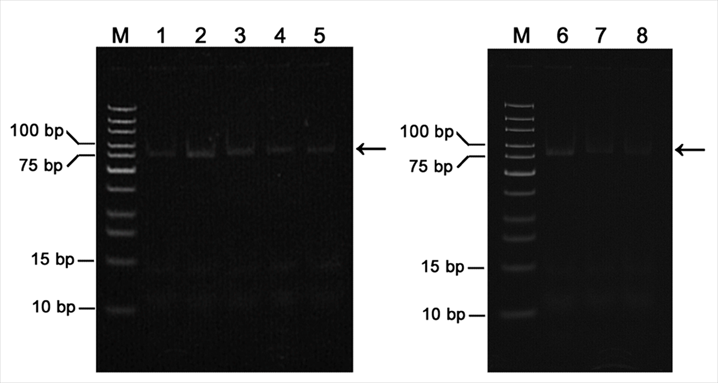

Supplement: S1 Fig — M, Ultra low range DNA ladder; lane 1, ath-miR-159a; lane 2, mmu-miR-122; lane 3, mmu-miR-21; lane 4, mmu-miR-20a; lane 5, mmu-miR-34a; lane 6, ath-miR-159a; lane 7, sja-miR-277; lane 8, sja-miR-3479-3p. (TIF) [file pntd.0003965.s001.tif]

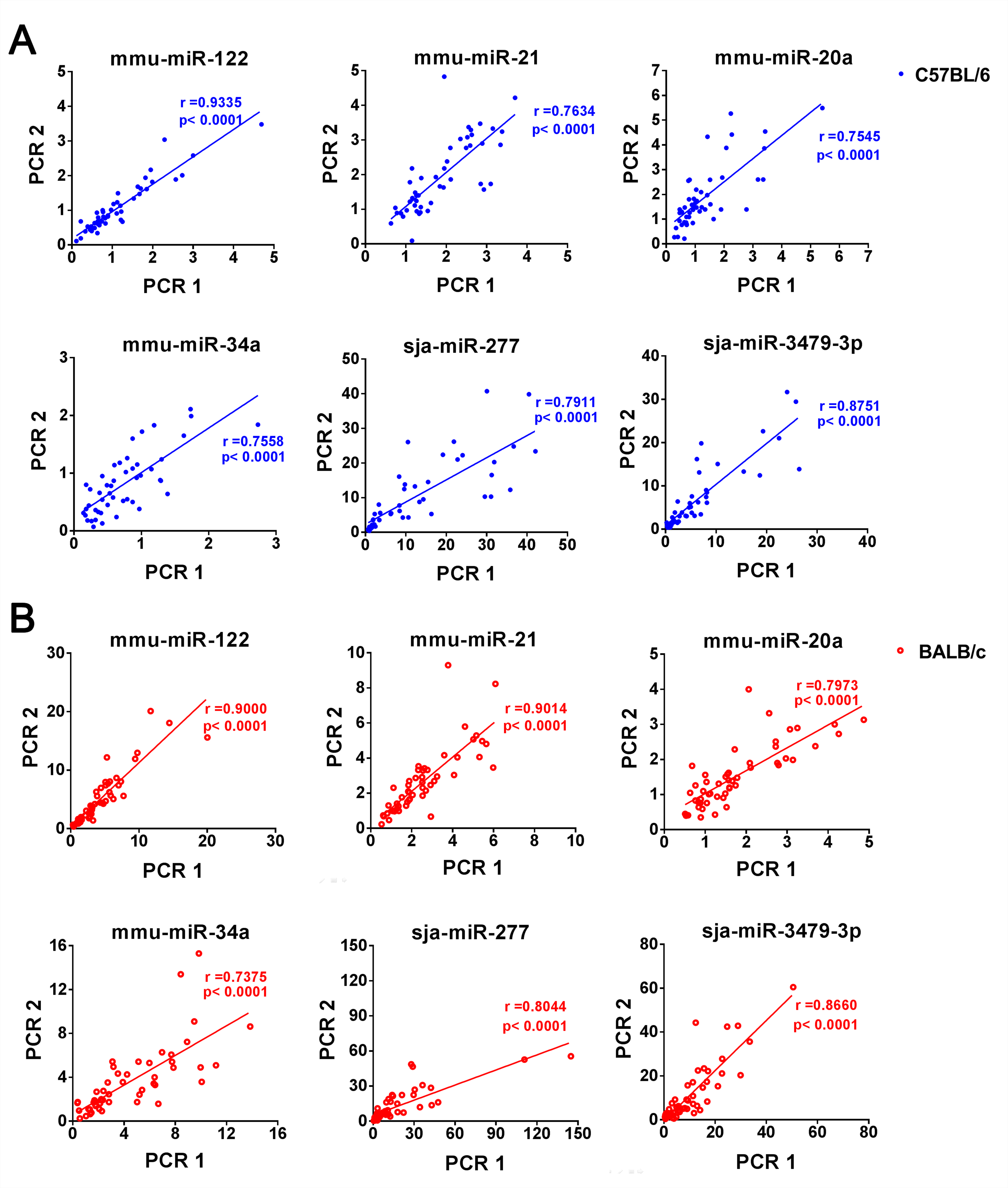

Supplement: S2 Fig — Pearson correlation values vary between 0.7375 and 0.9335. (TIF) [file pntd.0003965.s002.tif]

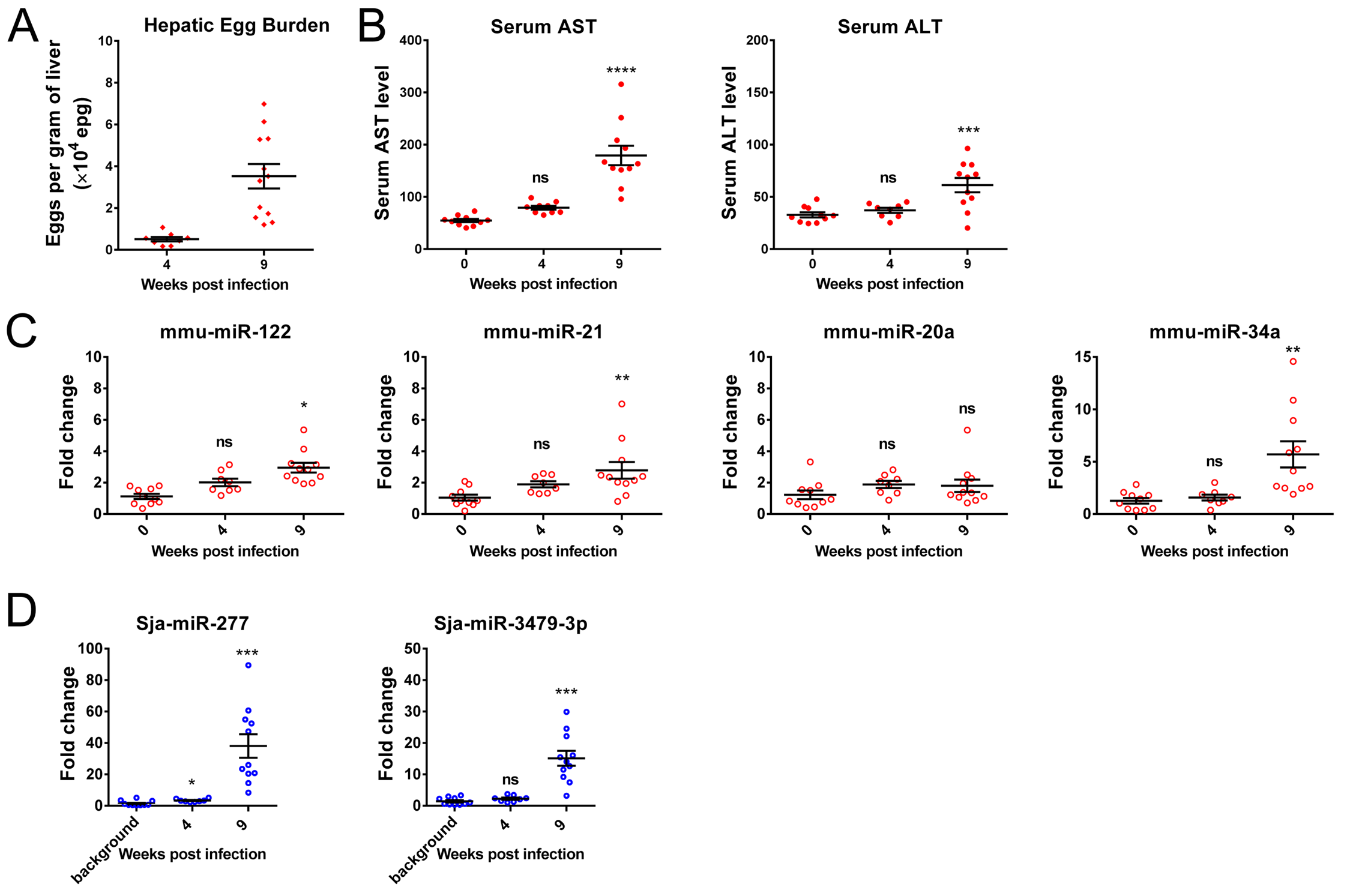

Supplement: S3 Fig — (A) Hepatic egg burdens; (B) Serum AST and ALT levels. Statistical significance between infected and naive mice was determined using 1-Way ANOVA (ns = no significant difference, *** = P<0.001, **** = P<0.0001). (C) Serum levels of host miRNAs (mmu-miR-122, mmu-miR-21, mmu-miR-20a and mmu-miR-34a); fold changes are defined as the ratio of serum miRNA abundance in infected mice compared with naive mice. Statistical significance between infected and naive mice was determined using 1-Way ANOVA (ns = no significant difference, * = P<0.05, ** = P<0.01); (D) Serum levels of parasite-derived miRNAs (sja-miR-277 and sja-miR-3479-3p); fold changes are defined as the ratio of miRNA abundance in the serum of infected mice compared with the background level in the serum of naive mice. Statistical significance between infected and naïve mice was determined using the Man-Whitney test (ns = no significant difference, * = P<0.05, *** = P<0.001). (TIF) [file pntd.0003965.s003.tif]

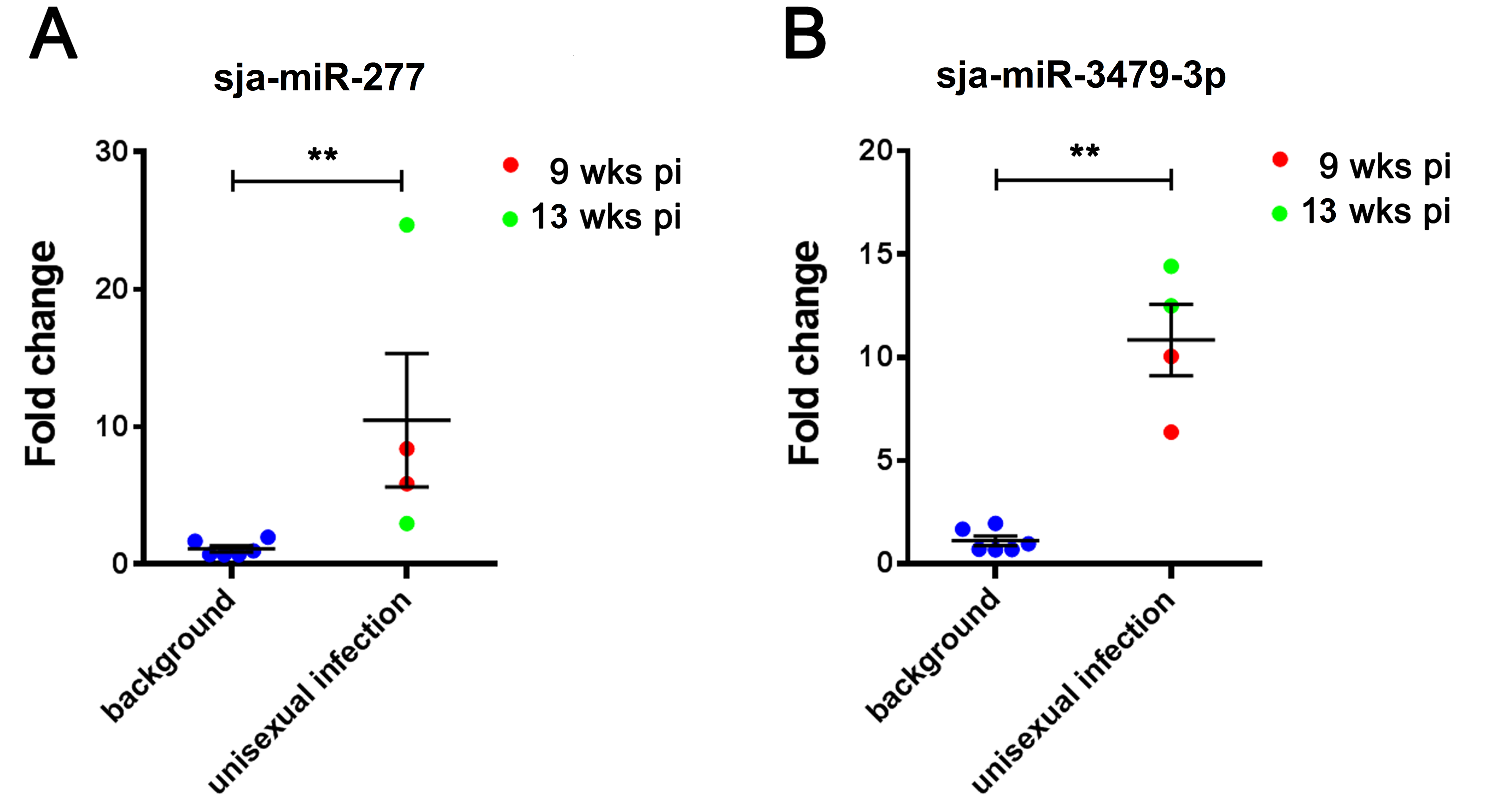

Supplement: S4 Fig — Fold changes are defined as the ratio of miRNA abundance in infected mice serum compared with the background abundance level in naive mice serum. Statistical significance between infected and naïve mice was determined using the Man-Whitney test (** = P<0.01). (TIF) [file pntd.0003965.s004.tif]
